# Risk Factors for Bloodstream Infections in Critically Ill Children: Gram‐Negative Predominance and Complex Chronic Conditions

**DOI:** 10.1111/apa.70268

**Published:** 2025-08-07

**Authors:** Vanessa Vicenzi, Ian Teixeira e Sousa, Jefferson Pedro Piva

**Affiliations:** ^1^ Postgraduate Program in Child and Adolescent Health Federal University of Rio Grande do Sul (UFRGS) Porto Alegre Rio Grande do Sul Brazil; ^2^ Federal University of Rio Grande do Sul (UFRGS) Porto Alegre Rio Grande do Sul Brazil

**Keywords:** aetiological agent, bloodstream infection, complex chronic conditions, mortality, paediatric intensive care, risk factors

## Abstract

**Aim:**

Bloodstream infections (BSIs) remain a significant cause of mortality in paediatric intensive care units (PICUs). This study aimed to identify risk factors associated with the aetiology and outcomes of hospital‐acquired BSIs.

**Methods:**

This retrospective study analysed 422 blood cultures with pathogenic growth collected from patients admitted to a PICU in Brazil between 2018 and 2021. Data on underlying conditions, central venous catheter and PICU length of stay were obtained through medical record review. Logistic regression assessed the association between BSI aetiology and mortality.

**Results:**

A total of 148 BSIs were analysed in children with a median age of 33.5 months [IQR 5–110]; 60.0% were male. Gram‐negative bacteria accounted for 43.2% of cases, Gram‐positive for 41.2% and fungi for 15.5%. Carbapenem resistance was observed in 26.5% of Gram‐negative isolates. Methicillin‐resistant *Staphylococcus aureus* represented 40.0% of *S. aureus* . Gram‐negative infections were associated with older age and oncohaematologic disease. Fungal infections were strongly associated with catheter use (OR 9.0; *p* = 0.039). Overall mortality was 24.3%. Vasoactive drug use was the only independent mortality predictor (RR 3.2; *p* = 0.004).

**Conclusions:**

Gram‐negative bacteria predominated and were associated with oncohaematologic disease. Recognising risk factors may improve empirical treatment in critically ill children.

**Abbreviations**
BSI: bloodstream infection
CCC: complex chronic condition
CI: confidence interval
CVC: central venous catheter
MRSA: methicillin‐resistant *Staphylococcus aureus*

OR: odds ratio
PICC: peripherally inserted central catheter
PICU: paediatric intensive care unit
PIM‐2: paediatric index of mortality 2
RR: relative risk

**Summary**

- Nosocomial bloodstream infections remain a relevant cause of morbidity and mortality in critically ill paediatric patients.
- This study identified Gram‐negative bacteria as the most frequent pathogens and their association with oncohaematologic conditions; fungal infections were linked to central venous catheter use.
- Recognising these risk factors can support earlier empirical treatment and may help reduce poor outcomes in children admitted to high‐complexity paediatric intensive care units.

## 1 Introduction

Bloodstream infections (BSIs) in paediatric patients are a significant cause of morbidity and mortality in intensive care settings, with some studies reporting attributable mortality as high as 80% [1, 2, 3, 4]. In addition, the association between BSIs and invasive devices has been well documented, particularly with central venous catheters (CVCs) [5, 6]. Paediatric intensive care units (PICUs) present a high degree of heterogeneity in the pathology profiles at admission [7]. This is due to the wide age range of patients and the types of diseases most frequently associated with specific age groups [7]. This diversity was amplified by the increased survival of patients with extreme prematurity, malignancies and complex chronic conditions (CCCs) [8, 9]. Furthermore, as approximately one‐fifth of patients with CCCs were technology dependent, the prevalence of children with CCCs admitted to PICUs was high, with reports ranging between 40% and 70% [8, 10].

The more severe the patient's underlying condition, the greater the likelihood of using invasive devices and greater the risk of developing a BSI [11]. Commonly evaluated risk factors, such as the duration of hospital stay, invasive devices, use of vasoactive drugs and mechanical ventilation, were often associated with nosocomial BSIs in both adult and paediatric populations [7]. Therefore, studies that also considered patient characteristics and comorbidities were more likely to predict the aetiology of BSIs [12]. With the emergence of patients with CCCs and increasingly dependent on PICU care, understanding the risk factors associated with the aetiology of BSIs and resistance patterns becomes essential. The aim of this study was to identify the association between common risk factors, CCC profiles and aetiological agents of BSIs in this case series.

## 2 Methods

### 2.1 Selection of Participants

This retrospective study evaluated 422 blood cultures with pathogenic growth collected from patients admitted to the PICU of Hospital de Clínicas de Porto Alegre, a referral centre in Southern Brazil, between 2018 and 2021. The hospital provides high‐complexity care for paediatric patients with rare genetic and oncologic diseases and serves as a national centre for paediatric liver and bone marrow transplantation. Patients who had blood cultures collected in other paediatric settings and were later transferred to the PICU were not included in the study.

The diagnostic criteria for defining BSIs followed the definitions established by the American Centers for Disease Control and Prevention [13]. A laboratory‐confirmed BSI was considered present if a pathogen not typically regarded as a contaminant was isolated in the absence of another infection site. Alternatively, BSI was also confirmed when a common contaminant was isolated in two separate blood cultures collected within 48 h, accompanied by clinical signs of sepsis. In the case of paired blood cultures from the same patient, only one result was considered to avoid duplication. Blood cultures with the same pathogen within 7 days were considered part of the same infection episode. All CVCs in this case series were assessed for potential catheter‐associated BSI, defined as a laboratory‐confirmed BSI in the presence of a catheter inserted for more than 2 days. The following microorganisms were considered contaminants: *Staphylococcus epidermidis* , *Staphylococcus hominis* , *Staphylococcus warneri* , *Staphylococcus haemolyticus* , *Staphylococcus capitis* , *Corynebacterium* spp., *Propionibacterium* spp., *Streptococcus viridans*, *Aerococcus* spp., *Micrococcus* spp., and *Bacillus* spp.

### 2.2 Data Collection and Measurements

Blood culture results were obtained from the clinical analysis laboratory of the hospital. Demographic and clinical data, including age, sex, reason for admission, laboratory parameters, presence of fever, resistance profile and Paediatric Index of Mortality 2 (PIM‐2), were obtained through medical record review. The PIM‐2 is a tool used to estimate the probability of mortality in patients admitted to the PICU, based on physiological and clinical variables collected within the first hour of admission. The score is converted into a percentage representing the predicted risk of death. Therefore, a PIM‐2 score of 15 corresponds to an estimated 15% mortality risk and is considered an indicator of high clinical severity. In the present study, this threshold was used to identify patients with increased clinical risk. The medical records were reviewed by two researchers (V.V. and I.T.S.), and in cases of doubt, the case was discussed with a senior researcher (J.P.P.). The following risk factors were assessed: presence of CCCs, duration of hospital and PICU stay, duration of mechanical ventilation, use of vasoactive drugs and duration of CVC use prior to blood culture. Patients with CCC were categorised as oncohaematologic disease, neurogenetic disease, liver disease, prematurity or others. The CCCs were grouped in a more summary way than the original categories as described by Feudtner et al. [14] to abbreviate the number of variables inserted in the multivariate analysis models. The study outcomes assessed in the presence of these risk factors were pathogen type and PICU mortality rate.

### 2.3 Statistical Analyses

Quantitative variables were described using means and standard deviations or medians and interquartile ranges. Categorical variables were described as frequencies and percentages. Comparisons between means were performed using Student's *t*‐test or analysis of variance. For non‐normally distributed variables, the Mann–Whitney *U* test or Kruskal–Wallis test was applied. Proportions were compared using the chi‐square or Fisher's exact tests. Risk analysis was conducted using Poisson regression, with univariate models followed by multivariate adjustments for confounding factors. Results were expressed as relative risk (RR), 95% confidence intervals (CI) and *p* values. For the fungal group, which had a smaller sample size compared to bacterial groups, a multinomial regression model was used, with results reported as odds ratios (OR). A *p* value < 0.05 was considered statistically significant. All analyses were performed using SPSS version 29.0.2.0 (International Business Machines Corporation, Armonk, New York, USA).

### 2.4 Ethics

The study was approved by the Ethics Committee of Hospital de Clínicas de Porto Alegre (CAAE 47154921700005327), and the requirement for informed consent was waived.

## 3 Results

### 3.1 Clinical Characteristics

Over the 36‐month study period, 2226 patients were admitted to the PICU, and 422 blood cultures obtained after admission showed microbial growth. After excluding duplicate samples and contaminants, 148 (35.0%) cases met the criteria for BSIs and were included in the analysis. The median age was 33.5 [IQR 5–110] months, and 60.0% were male (Table 1). Upon admission, the median PIM‐2 was 2.6 [IQR 0.9–7.1], and the most frequent diagnoses leading to PICU transfer were sepsis (45.3%), respiratory dysfunction (20.0%) and hepatic disorder (8.0%). Other diagnoses that did not fall under the category of major organ dysfunction were categorised as other (14.8%).

**TABLE 1:** Characteristics of paediatric patients with bloodstream infections admitted to the PICU from January 2018 to January 2021.

Patients, *n*	128
Age (months)	33.5 [IQR 5–110]
Gender (male), *n* (%)	90 (60.0%)
PIM—2 (%)	2.55 [IQR 0.9–7]
CCC, *n* (%)	
Healthy	15 (10.0%)
Neurological disorder	12 (8.0%)
Hepatic disorder	11 (7.4%)
Malignancies	32 (21.6%)
Respiratory	1 (0.7%)
Liver transplant	13 (8.8%)
Bone marrow transplant	6 (4.0%)
Prematurity	16 (10.8%)
Genetic disorder	22 (14.8%)
Haematological disorder	2 (1.4%)
Other	18 (12.0%)
Reason for admission, *n* (%)	
Haematological	2 (1.4%)
Respiratory	30 (20%)
Neurological	7 (4.7%)
Infection	67 (45.3%)
Cardiological	6 (4.0%)
Postoperative	10 (6.7%)
Hepatic disorder	12 (8.0%)
Transfer from neonatology	6 (4.0%)
Presence of fever, *n* (%)	87 (58.8%)
Use of vasoactive agents, *n* (%)	66 (45.0%)
Use of central catheter, *n* (%)	122 (82.4%)
Duration of central catheter (days)	12 [IQR 4–33]
Mechanical ventilation, *n* (%)	63 (42.5%)
Duration of MV (days)	4 [IQR 0–11.5]
Death, *n* (%)	36 (24.3%)
Bacterial profile, *n* (%)	
Gram‐negative Bacilli	64 (43.2%)
*Pseudomonas* sp.	19 (29.7%)
*Acinetobacter*	4 (6.3%)
*Staphylococcus aureus*	20 (13.5%)
MRSA	8 (40.0%)
Coagulase‐negative *Staphylococcus*	22 (14.8%)
*Enterococcus* sp.	13 (8.8%)
*Streptococcus viridans*	4 (2.7%)
*S. pneumoniae*	2 (1.4%)
*Streptococcus* sp.	1 (0.7%)
*Haemophilus* sp.	1 (0.7%)
*Candida albicans*	7 (4.7%)
Non‐*albicans Candida*	13 (8.8%)
Others	7 (4.7%)

*Note:* All values are described as median [IQR] or absolute value (%).

Abbreviations: CCC, complex chronic condition; MRSA, methicillin‐resistant *Staphylococcus aureus*; MV, mechanical ventilation; PIM‐2, Paediatric Index of Mortality.

Complex chronic conditions were present in 90.0% of the cases. The most frequent CCC was malignancy (21.6%), followed by genetic diseases (14.8%) and organ and tissue transplants (12.8%). At the time of the blood culture collection, 45.0% were using vasoactive drugs and 59.0% had a documented fever in the previous 24 h. CVCs were present in 82.4% of cases, with a median duration of 12 days [IQR 4–33] prior to infection. The insertion sites were the jugular vein (53.0%), subclavian vein (24.0%) and femoral vein (4.0%). Additionally, nine cases used peripherally inserted central catheters (PICC), six had Port‐a‐Cath devices and one had a Broviac catheter. Mechanical ventilation was used at the time of infection in 42.5% of patients, with a median of 4 days [IQR 0–11.5] of prior ventilation.

### 3.2 Pathogens

Gram‐negative bacteria were identified in 43.2% of cases, Gram‐positive bacteria in 41.2% and fungi in 15.5%. Among the Gram‐negative bacteria, *Pseudomonas* species were present in 29.7% of samples, and *Acinetobacter* species in 6.3%. Carbapenem resistance was observed in 26.5% of Gram‐negative isolates, of which 82.0% were carbapenem‐resistant *Pseudomonas*. Multidrug resistance occurred in 29.6% of Gram‐negative bacteria. Among Gram‐positive bacteria, *Staphylococcus* species accounted for 14.8% of all BSIs, and *Staphylococcus aureus* for 13.5%. Of the *S. aureus* isolates, 40.0% were methicillin resistant (MRSA). Among the fungal infections, 65% of cases were due to non‐*albicans Candida* species, with only one case of fluconazole resistance.

Children with Gram‐negative infections had a higher median age of 85.5 months [IQR 11–132] compared to the other groups (*p* < 0.001). In the Gram‐negative group, oncohaematologic disease was the most frequent CCC (47.0%), followed by neurogenetic diseases (26.0%). A similar trend was seen in the fungal group, with 26.0% presenting oncohaematologic disease and 17.0% neurogenetic conditions. Among Gram‐positive infections, neurogenetic disease was most common (23.0%), followed by prematurity‐related sequelae (21.0%). Oncohaematologic disease was associated with a fourfold higher risk of Gram‐negative BSI compared to Gram‐positive infection (RR 4.1; 95% CI 1.5–11.3) (Table 2).

**TABLE 2:** Risk factors for Gram‐negative bloodstream infections.

	Gram‐negative	Gram‐positive	Univariate analysis	Multivariate analysis
*n* (125)	64 (51.2%)	61 (48.8%)		
Complex chronic condition, *n* (%)				
Healthy	3 (4.6%)	11 (18.0%)	RR = 1	RR = 1
Oncohaematologic disease	30 (47.0%)	4 (6.5%)	RR 4.1 (95% CI 1.4–11.3)	RR 3 (95% CI 1.1–8.3)
Neurogenetic disease	17 (26.0%)	14 (23.0%)	RR 2.5 (95% CI 0.8–7.3)	RR 2.3 (95% CI 0.8–6.4)
Hepatic disease	9 (14.0%)	8 (13.0%)	RR 2.4 (95% CI 0.8–7.4)	RR 2.3 (95% CI 0.7–6.9)
Prematurity	1 (1.5%)	13 (21.0%)	RR 0.3 (95% CI 0.2–2.8)	RR 0.3 (95% CI 0.4–2.3)
Other	4 (6.2%)	11 (18.0%)	RR 1.2 (95% CI 0.3–4.6)	RR 1.2 (95% CI 0.3–4.3)
Age (months)	85.5 [IQR 11–132]	13 [IQR 3–37]	*p* < 0.001 RR 1.005 (95% CI 1.002–1.007)	*p* = 0.41 RR 1.001 (95% CI 0.99–1.004)
PIM 2	4.6 [IQR 1.2–8.6]	1.3 [IQR 0.9–4.2]	*p* = 0.10 RR 1.012 (95% CI 0.99–1.02)	—
PIM 2 > 15, *n* (%)	11 (17.0%)	2 (3.0%)	*p* < 0.001 RR 1.7 (95% CI 1.3–2.4)	*p* < 0.001 RR 1.5 (95% CI 1.2–2)
CVC on the day of blood drawing, *n* (%)	58 (90.0%)	42 (69.0%)	*p* = 0.016 RR 2.4 (95% CI 1.1–4.9)	*p* = 0.42 RR 1.3 (95% CI 0.6–2.8)
Blood culture site				
Central	31 (48.0%)	22 (36.0%)	*p* = 0.158 RR 1.2 (95% CI 0.9–1.7) RR = 1	—
Peripheral	33 (52.0%)	39 (64.0%)		
Prior duration of CVC (days)	15 [IQR 4.8–40.3]	6 [IQR 3–14]	*p* = 0.003 RR 1.001 (95% CI 1–1.001)	—
MV on the day of blood drawing, *n* (%)	27 (42.0%)	24 (39.0%)	*p* = 0.745 RR 1.05 (95% CI 0.7–1.4)	—
Prior duration of MV (days)	1 [IQR 0–13.5]	5.5 [IQR 0–15]	*p* = 0.167 RR 1.001 (95% CI 1–1.002)	—
Vasoactive drugs on the day of blood drawing, *n* (%)	36 (56.2%)	20 (32.8%)	*p* = 0.009 RR 1.5 (95% CI 1.1–2.2)	*p* = 0.35 RR 1.1 (95% CI 0.8–1.5)
Prior PICU stay (days)	0 [IQR 0–6]	1 [IQR 0–9]	*p* < 0.001 RR 1.001 (95% CI 1.001–1.002)	*p* = 0.21 RR 1.001 (95% CI 1.000–1.002)
Prior hospital stay (days)	14 [IQR 1–34]	11 [IQR 1–34]	*p* = 0.55 RR 1 (95% CI 0.99–1.002)	—

*Note:* All values are described as median [IQR] or absolute value (%). RR (95% CI) is used to express risk. A 95% confidence interval (*p* < 0.05) was used. Variables included in the model using the Poisson regression method: PIM‐2 above 15%, age, presence of CVC on the day of the exam, use of vasoactive drugs, prior PICU stay and comorbidities.

Comparison of PIM‐2 between the bacterial groups indicated that 17.0% of patients with Gram‐negative bacteria had an estimated mortality risk above 15% compared to 3.0% in the Gram‐positive group (*p* < 0.001).

No differences were observed in the length of hospital stay prior to PICU admission between groups. Central venous access was present in 90.0% of patients with Gram‐negative bacteria and in 42.0% of those with Gram‐positive infections (*p* = 0.016), representing a 2.5‐fold higher risk. The median duration of CVC use prior to blood culture collection was longer in the Gram‐negative group: 15.0 days [IQR 4.8–40.3] versus 6.0 days [IQR 3.0–14.0] in the Gram‐positive group (*p* = 0.003).

The use of mechanical ventilation at the time of blood culture and the duration of prior mechanical ventilation did not differ significantly between the groups. However, vasoactive drugs at the time of culture collection represented a 1.5‐fold higher RR of Gram‐negative infection (*p* = 0.009). Based on these findings, a multivariate analysis was performed to identify the independent predictors of Gram‐negative BSI. After adjusting for confounders, oncohaematologic disease, PIM‐2 score above 15% and longer PICU stay prior to culture remained statistically significant predictors. Oncohaematologic disease conferred a threefold higher risk of developing a Gram‐negative BSI, while PIM‐2 above 15% corresponded to a RR of 1.6 (95% CI 1.2–2.1). Each additional day in the PICU before blood culture collection was associated with a cumulative daily increase in risk of 0.1% (RR 1.001; 95% CI: 1.000–1.002).

The same risk factors were evaluated for the fungal group. In the multinomial regression model, which included PIM‐2 > 15.0%, age, CVC presence on the day of culture, vasoactive drug use and comorbidities, only the presence of a CVC remained independently associated with fungal infection (OR 9.0; 95% CI 1.1–72.5; *p* = 0.039), indicating an approximately ninefold increased risk.

### 3.3 Mortality

The overall mortality rate in this case series was 24.3%, with Gram‐negative infections accounting for 61.0% of deaths (Table 3). Considering the Gram‐positive group as the reference, Gram‐negative infections were associated with a threefold higher risk of death and fungal infections with a 2.5‐fold higher risk. The median age of patients who died was 98.5 months [IQR 38–133.5] compared to 21 months [IQR 4.0–87.2] among survivors (*p* < 0.001). Oncohaematologic disease was the most frequent CCC among deceased patients, conferring a RR of 4.3 (95% CI: 1.2–16.1). Invasive device use differed between survivors and non‐survivors. The use of mechanical ventilation and vasoactive drugs at the time of blood culture collection was significantly higher among those who died (*p* = 0.012 and *p* < 0.001, respectively). For Gram‐positive infections, each additional day of CVC use before the blood culture was associated with a 1.8% daily increase in the risk of death. Independent predictors of mortality were assessed using a Poisson regression model including pathogen group, CCC, age, PIM‐2 score above 15%, presence of CVC on the day of culture and use of vasoactive drugs. Only the use of vasoactive drugs remained statistically associated with death, with a RR of 3.3 (95% CI: 1.5–7.3; *p* = 0.004), indicating an approximately threefold higher risk of death.

**TABLE 3:** Risk factors for death.

	Survivors	Deaths	Univariate analysis	Multivariate analysis
*n* (148)	112 (75.6%)	36 (24.3%)		
Pathogen				
Gram‐positive	54 (48.2%)	7 (19.4%)	RR = 1	RR = 1
Gram‐negative	42 (37.5%)	22 (61.0%)	RR 2.9 (95% CI 1.3–6.5)	RR 1.3 (95% CI 0.5–3.1)
Fungi	16 (14.2%)	7 (19.4%)	RR 2.6 (95% CI 1.0–6.7)	RR 1.6 (95% CI 0.6–4.1)
Complex chronic condition, *n* (%)				
Healthy	13 (11.6%)	2 (5.5%)	RR = 1	RR = 1
Oncohaematologic disease	17 (15.2%)	23 (63.8%)	RR 4.3 (95% CI 1.1–16.1)	RR 2.7 (95% CI 0.7–9.9)
Neurogenetic disease	32 (28.5%)	3 (8.3%)	RR 0.6 (95% CI 0.1–3.4)	RR 0.5 (95% CI 0.1–2.7)
Hepatic disease	19 (17.0%)	4 (11.1%)	RR 1.3 (95% CI 0.2–6.2)	RR 1.5 (95% CI 0.3–6.3)
Prematurity	12 (10.7%)	4 (11.1%)	RR 1.8 (95% CI 0.4–8.7)	RR 2 (95% CI 0.5–8)
Other	19 (17.0%)	0 (0.0%)	—	—
Age (months)	21 [IQR 4–87.2]	98.5 [IQR 38–133.5]	*p* < 0.001 RR 1.008 (95% CI 1.004–1.012)	*p* = 0.63 RR 1.001 (95% CI 0.99–1.007)
PIM 2	1.35 [IQR 0.8–4.32]	8.2 [IQR 4.65–9.42]	*p* < 0.001 RR 1.03 (95% CI 1.019–1.042)	—
PIM 2 > 15, *n* (%)	8 (7.1%)	8 (22.2%)	*p* = 0.004 RR 2.3 (1.3–4.2)	*p* = 0.82 RR 1.0 (95% CI 0.6–1.8)
CVC on the day of blood drawing, *n* (%)	83 (74.1%)	34 (94.4%)	*p* = 0.064 RR 3.6 (95% CI 0.9–14.1)	*p* = 0.97 RR 0.9 (95% CI 0.2–4.6)
Blood culture site				
Central				
Gram‐positive	21 (42.0%)	1 (6.5%)	RR = 1	—
Gram‐negative	21 (42.0%)	10 (66.0%)	RR 7 (95% CI 0.9–51.4), *p* = 0.053	
Fungi	8 (16.0%)	4 (27.5%)	RR 7.3 (95% CI 0.9–58.4), *p* = 0.06	
Peripheral				
Gram‐positive	33 (53.0%)	6 (29.0%)	RR = 1	*—*
Gram‐negative	21 (34.0%)	12 (57.0%)	RR 2.3 (95% CI 0.9–5.6), *p* = 0.051	
Fungi	8 (13.0%)	3 (14.0%)	RR 1.7 (95% CI 0.5–5.9), *p* = 0.35	
Prior duration of CVC (days)	8 [IQR 3–26.5]	19.5 [IQR 10–86]	*p* = 0.35 RR 1.001 (95% CI 0.99–1.002)	—
Prior duration of CVC by pathogens (days)				
Gram‐positive	5 [IQR 2.5–12.5]	11.5 [IQR 6.75–23]	RR 1.018 (95% CI 1.001–1.036), *p* = 0.038	*p* = 0.22
Gram‐negative	11 [IQR 3.5–32.5]	20 [IQR 13–97]	RR 1 (95% CI 0.99–1.001), *p* = 0.89	
Fungi	20 [IQR 12–39]	30 [IQR 8–136]	RR 1.002 (95% CI 0.99–1.005), *p* = 0.22	
MV on the day of blood drawing, *n* (%)	41 (36.6%)	22 (61.1%)	*p* = 0.012 RR 2.1 (95% CI 1.1–3.8)	*p* = 0.32 RR 1.2 (95% CI 0.7–2)
Prior duration of MV (days)	1 [IQR 0–7]	8.5 [IQR 0.25–14.5]	*p* = 0.448 RR 0.99 (95% CI 0.99–1.003)	—
Vasoactive drugs on the day of blood drawing, *n* (%)	37 (33.0%)	29 (80.0%)	*p* < 0.001 RR 5.1 (95% CI 2.4–10.9)	*p* = 0.004 RR 3.2 (95% CI 1.4–7.2)

*Note:* All values are described as median [IQR] or absolute value (%). RR (95% CI) is used to express risk. A 95% confidence interval (*p* < 0.05) was used. Variables included in the model using the Poisson regression method: pathogens, PIM above 15%, age, presence of CVC on the day of the exam, use of vasoactive drugs, MV on the day of blood drawing and comorbidities.

## 4 Discussion

This study showed that 6.6% of patients admitted to the PICU developed BSIs, corresponding to a rate of 66 episodes per 1000 admissions. Similar rates were reported in studies of patients with comparable characteristics [3]. The main risk factors for paediatric BSIs included the use of invasive devices such as CVCs, urinary catheters and feeding tubes. Additional factors were prolonged PICU stay, mechanical ventilation, vasoactive drug use and immunosuppressive conditions [1, 7]. In this regard, the high prevalence of patients with CCC was notable in this case series, reaching 90.0%. The dependence on technology for life support in this population has been described as around 20% [8]. After malignancy, which was also a frequent comorbidity in other services [8, 15], the second and third most common conditions in this sample were organ and tissue transplantation, followed by genetic diseases.

The increase in PICU admissions of children with CCCs and the varying rates of BSIs have been described in other studies [12, 16]. Borensztajn et al. [17] reported that 16% of patients who sought care in paediatric emergency departments had CCCs. These patients had a fivefold higher chance of PICU admission and a threefold higher likelihood of blood culture collection compared to previously healthy patients. Once in the PICU, the proportion of patients with CCCs requiring invasive procedures and technological support could reach 90% [10].

Central venous catheter use in this case series was 82.4%, which was comparable to other services and characteristic of this population [3, 7]. Even with this high frequency of CVC use, the rate of catheter‐related BSIs was 44.0%, lower than the rates of up to 70% described in other high‐complexity centres [6, 18, 19]. When analysed as a risk factor, the presence of a CVC was associated with the development of Gram‐negative infections in univariate analysis, as well as the duration of catheter use prior to the infection. The median duration observed was similar to that in previous studies [3]. The contamination rate in blood cultures, defined by the growth of usual contaminants, was 65.0%, consistent with previously documented rates [12, 20].

Improvements in bacterial identification techniques and prevention programmes have contributed to a better characterisation of the aetiological agents of BSIs [6]. In BSIs not associated with catheters, Gram‐negative bacteria were also the predominant pathogens in this study. Given their significant morbidity and mortality impact [21, 22], the identification of risk predictors for these pathogens was a focus of this analysis. Among the established categories of CCC, oncohaematologic disease was independently associated with Gram‐negative BSIs, with a threefold higher risk.

The aetiological distribution of catheter‐related BSIs may have been influenced by the predominance of patients with CCCs, particularly those with malignancy. Studies have reported a growing prevalence of coagulase‐negative *Staphylococcus* and fungal infections as leading causes of catheter‐related BSIs [23]. However, in immunosuppressed patients, Gram‐negative bacteria still prevail as the main aetiological agents [18]. Although Gram‐negative bacteria were the most common pathogens, the rates of multidrug‐resistant strains (30.0%) and carbapenem resistance (26.5%) were lower than previously reported [3, 7]. Considering the high complexity of the population, the relatively low incidence of hospital‐acquired pathogens such as *Pseudomonas* species and *Acinetobacter* species, compared to other locations, was noteworthy [7, 24].

A significant finding of this study was the MRSA prevalence of 40.0% among *S. aureus* isolates. Similar rates have been reported in some developing countries [23, 25], although other studies, such as Deniz et al. [7], documented a 100% methicillin resistance rate among *S. aureus* BSIs.

In addition to the increase in Gram‐negative BSIs, other studies have shown the emergence of fungi as aetiological agents of BSIs [26]. In this study, the fungal infection rate was 15.5%, with 65.0% being non‐*albicans Candida* species and a low rate of fluconazole resistance. Comparatively, Deniz et al. [7] reported a 33% prevalence of BSIs caused by fungi, with 47% of cases due to non‐*albicans Candida* species and a fluconazole resistance rate of 23%. The use of multinomial regression identified the presence of a CVC as the only independent factor associated with fungal infection (OR 9.0; 95% CI 1.1–72.5; *p* = 0.039), consistent with findings from other studies [7, 27].

Knowledge of the pathogenic profile in high‐complexity services directly influences mortality. Aksoy et al. [18] reported that inappropriate empirical antibiotic therapy in BSIs was directly associated with increased mortality in immunosuppressed patients [18]. The overall ICU mortality in this case series was 24.3%, comparable to the 25.0% reported in the Sepsis Prevalence, Outcomes, and Therapies (SPROUT) study, which included paediatric patients with septic shock [28]. Similar rates were found by Rech et al. [29], who analysed sepsis in patients with CCCs, reporting a mortality rate of 22.0% [20, 29]. The incidence of sepsis is higher in CCC patients, and the diagnosis is associated with higher mortality in this group [29]. Approximately 60% of paediatric patients with septic shock require mechanical ventilation and vasoactive drugs, both associated with higher mortality rates [16]. Among the CCC categories, oncohaematologic disease was associated with an increased risk of death. Additionally, Gram‐negative and fungal BSIs were linked to higher mortality, as well as a 1.8% daily cumulative risk of death in patients with Gram‐positive catheter‐related infections. However, in the multivariate analysis, the use of vasoactive drugs at the time of blood culture collection remained the only independent risk factor, increasing mortality risk by approximately threefold (RR 3.3; 95% CI 1.5–7.3; *p* = 0.004). This association underscores the critical condition of these patients, as vasopressor dependence reflects haemodynamic instability and multiorgan dysfunction.

### 4.1 Strengths and Limitations

This single‐centre study featured a distinctive patient population characterised by pronounced clinical heterogeneity and a predominance of CCCs, which may limit the generalisability of the findings. The distribution of CCCs may have introduced bias, considering that oncohaematologic diseases and transplant recipients constituted two of the main categories. These conditions include prolonged hospital stays, multiple invasive devices and immunosuppression inherent to the diagnosis, all of which were reflected in the prevalence of BSIs and the outcomes analysed. Moreover, the exclusion of patients whose blood cultures were collected in other hospital units prior to PICU admission for complications may have inadvertently selected against cases with fewer comorbidities and reduced use of invasive devices. The decision to use the PIM‐2 to characterise the study sample was based on our setting as a general PICU with high‐complexity care in a low‐income country, where PIM‐2 has been validated as a reliable tool for assessing mortality risk. However, relying solely on PIM‐2 may represent a limitation, as the independent association of CCCs with PICU mortality suggests that many chronic conditions confer risks beyond those predicted by this score. Despite these limitations, the study provided relevant insights into risk factors, the profile of infecting microorganisms and antimicrobial resistance in this high‐risk population. Although the distribution of conditions was uncommon, the findings were comparable to other studies involving similar populations.

## 5 Conclusion

This study found that BSIs remain a significant diagnostic challenge associated with high morbidity and mortality in the PICU setting. The increased survival of patients with CCCs has made this population increasingly dependent on specialised healthcare services. Despite the implementation of care bundles and preventive measures, the incidence and mortality rates of nosocomial BSIs remain high. A better understanding of the risk factors associated with BSIs in this emerging population is essential to guide individualised and optimised treatment strategies.

## Conflicts of Interest

The authors declare no conflicts of interest.

## Data Availability

The data that support the findings of this study are available from the corresponding author upon reasonable request.
